# Positive Lymph Node Metastasis Has a Marked Impact on the Long-Term Survival of Patients with Hepatocellular Carcinoma with Extrahepatic Metastasis

**DOI:** 10.1371/journal.pone.0095889

**Published:** 2014-04-23

**Authors:** Feng Xia, Lin Wu, Wan-Yee Lau, Guo Li, Hongbo Huan, Cheng Qian, Kuansheng Ma, Ping Bie

**Affiliations:** 1 Institute of Hepatobiliary Surgery and Southwest Cancer Center, Southwest Hospital, Third Military Medical University, Chongqing, China; 2 Faculty of Medicine, The Chinese University of Hong Kong, Shatin, Hong Kong SAR, China; 3 Institute of Pathology and Southwest Cancer Center, Southwest Hospital, Third Military Medical University, Chongqing, China; Xiangya Hospital of Central South University, China

## Abstract

**Background:**

The prognosis of hepatocellular carcinoma (HCC) patients with extrahepatic metastasis is extremely poor. However, what is the main risk factor for survival remains unclear for these patients. We aimed to find out the relative frequency, incidence and locations of extrahepatic metastases and the risk factors of long-term survival of the patients.

**Methods:**

132 HCC patients with extrahepatic metastasis diagnosed by ^18^F-FDG PET/CT and conventional workup were enrolled into this study. The incidence and locations of extrahepatic metastases were summarized, and the related risk factors of overall survival were analyzed.

**Results:**

The most frequent extrahepatic metastatic sites were lymph nodes in 72 (54.5%), bone in 33 (25.0%) and lung in 28 (21.2%) patients. On univariate analysis, prothrombin time, Child-Pugh grade, portal/hepatic vein invasion and lymph node metastasis were independent risk factors of overall survival. On multivariate analysis, lymph node metastasis was the only independent risk factor of overall survival. The cumulative survival rates at 1- and 3-years after diagnosis of extrahepatic metastasis of HCC were 34.4% and 9.3%, respectively. The median survival time was 7 months (range 1 ∼38 months). The median survival time for patients with or without lymph node metastasis were 5 months (range 1∼38 months) and 12 months (range 1∼30 months), respectively (*P* = 0.036).

**Conclusions:**

This study showed lymph nodes to be the most frequent site of extrahepatic metastases for primary HCC. Lymph node metastasis was the main risk factor of overall survival in patients with HCC with extrahepatic metastasis.

## Introduction

Hepatocellular carcinoma (HCC) is the third commonest cause of cancer-related death worldwide [1]. Its prognosis is related to the tumor stage and to the patient's underlying liver function. As extrahepatic metastases are present in 13.5–42% of patients at the time of diagnosis [2]–[4], adequate pretreatment staging of HCC is important [5]–[7].

Positron emission tomography/computed tomography with 18F-fluorodeoxyglucose (^18^F-FDG PET/CT) is useful for diagnosis, staging, and monitoring treatment response in patients with liver neoplasms [8]–[13]. It is more powerful in detecting extrahepatic metastases of primary HCC than the conventional imaging methods using ultrasonography (US), computed tomography (CT), and magnetic resonance imaging (MRI) [6], [13]. Recently, a systematic review and meta-analysis concluded that ^18^F-FDG PET/CT has good diagnostic performance on metastatic or recurrent HCC, with significantly better sensitivity and specificity in detecting metastatic HCC than the conventional imaging methods [14].

The prognosis of patients with HCC with extrahepatic metastasis is extremely poor. Although common sites of extrahepatic metastases of HCC using CT and autopsy studies have been reported [15]–[17], the role of ^18^F-FDG PET/CT in the detection of these metastases is still unclear. This study was conducted to find out in patients with HCC, the relative frequency, incidence and locations of extrahepatic metastases using ^18^F-FDG PET/CT. The risk factors of long-term survival of patients with extrahepatic metastases would also be determined using univariate and multivariate analyses on clinicopathological data.

## Patients and Methods

All patients gave written informed consent to this study which had been approved by the Institutional Review Board of the Southwest Hospital, Third Military Medical University. Between August 2003 and November 2011, consecutive patients who were diagnosed to have HCC according to the diagnostic criteria of the European Association for the Study of the Liver were routinely evaluated by ^18^F-FDG PET/CT. A diagnosis of HCC was made when two different imaging examinations revealed typical features of HCC in a patient with an elevated α-fetoprotein level (≥400 ng/mL) or when there was a histologic diagnosis of HCC.

### ^18^F-FDG PET/CT procedure and Interpretation

The ^18^F-FDG PET/CT (Discover LS; General Electric Medical Systems, Milwaukee, WI) examinations were carried out using a standard protocol. The patient was fasted for at least 6 hours before imaging. Serum glucose levels measured at the time of ^18^F-FDG injection were <130 mg/dL in all the patients. A low-dose non-contrast CT scan was obtained first for attenuation correction. Then, a transverse emission scan was initiated from proximal feet to head with five bed positions about 1 hour after administration of 320–450 MBq of ^18^F-FDG. The attenuation-corrected ^18^F-FDG PET/CT images were interpreted by the consensus of two or more experienced nuclear medicine specialists who were unaware of the clinical information. The standardized uptake value (SUV) was assessed by the region of interest (ROI), which was drawn over the areas of maximum intensity in each lesion. A positive malignant ^18^F-FDG uptake was defined as an abnormal increase in comparison with the background activity in a normal contralateral structure or surrounding tissues, and vice versa. A final decision was made by consensus on evaluation of the results of the ^18^F-FDG PET/CT and characteristics of conventional workup.

### Statistical Analysis

Statistical analysis was performed using the chi-square test, Mann-Whitney U test or the Fisher exact test to compare discrete variables. The overall survival was defined as the time interval from the date of diagnosis to the date of death from any cause or to the last visit before the date of censor of this study on November 30, 2011. The survival rate and the mean survival time were estimated by the Kaplan-Meier survival analysis. Difference in survival between the groups was assessed by the log-rank test. All the statistical analysis was performed with SPSS 13.0 for Windows (SPSS Inc., Chicago, IL, USA), and a *P* value <0.05 was considered statistically significant.

## Results

Of 451 patients who were diagnosed to have HCC during the study period, 132 were found to have extrahepatic metastases (29.3%). There were 104 men and 28 women. The median age was 48 years (range 26–86 years). The median follow-up period was 26 months (range 6–100 months). Table 1 summarizes the clinical data of these 132 patients.

**Table 1:** Characteristics of HCC patients.

Variables	n = 132
Sex (Male/Female)	104/28
Age (years) *	48(26∼86)
Etiology(HBV/others)	105/27
Performance status (PS)(0/1/2/3/4)	69/52/8/2/1
Serum albumin (g/L) *	39.6(24∼54.6)
Total bilirubin(µmol/L) *	16.3(5.6∼338.2)
Prothrombin time (s)	12.7(10.4∼23.9)
AFP level (ng/mL) *	12.3(0.2∼1123156.0)
Child–Pugh grade(A/B/C)	108/20/4
Portal/hepatic vein invasion(Yes/No)	51/81
CLIP Score(0/1/2/3/4/5/6)	5/12/18/52/32/11/2
TNM stage(III/IV)	5/127
BCLC stage(C/D)	126/6

*Value expressed in median with range in parentheses.

### Sites of Extrahepatic Metastases

The sites of extrahepatic metastases of HCC are summarized in Table 2. There were 165 extrahepatic metastatic sites in 132 HCC patients. Eight patients had widespread extrahepatic metastases all over the body. The most frequent extrahepatic metastatic sites were lymph nodes in 72 (54.5%) patients, bone in 33 (25.0%) patients and lung in 28 (21.2%) patients.

**Table 2:** Site of extrahepatic metastases in 132 HCC patients with extrahepatic metastases.

Sites	Patients (n = 132), n (%)
Lymph nodes	72(54.5)
Retroperitoneal lymph node	32(24.2)
Porta hepatis lymph node	15(11.4)
Mediastinal lymph node	13(9.8)
Paraaortic lymph node	9(6.8)
Periceliac lymph node	9(6.8)
Cervical lymph node	6(4.5)
Infraclavicular lymph node	6(4.5)
Others*	7(5.3)
Bone	33(25.0)
Thoracic vertebrae	9(6.8)
Ilium	9(6.8)
Ribs	5(3.8)
Lumbar vertebrae	4(3.0)
Sternum	4(3.0)
Others**	18(13.6)
Lung	28(21.2)
Peritoneum	7(5.3)
Pancreas	5(3.8)
Pleura	5(3.8)
Stomach	4(3.0)
Others***	11(8.3)

*including Submandibular, Mesenteric, Nasal, Iliac, Axillary and Retroauricular lymph node

**including Sacrum, Skull, Clavicle, Femur, Cervical vertebrae, Scapula, Ischium and Humerus

***including Adrenal glands, Diaphragm, Limbs, Spleen, Bladder, Brain and Duodenum.

The 72 patients with extrahepatic lymph node metastases involved 97 metastatic lymph nodes. These metastatic lymph nodes were characterized by increase in size with positive ^18^F-FDG uptake. Seven patients had widespread lymph node metastases. The most common metastatic lymph nodes were the retroperitoneal lymph nodes in 32 (24.2%) patients, followed by the porta hepatis lymph nodes and the mediastinal lymph nodes in 15 (11.4%) and 13 (9.8%) patients, respectively. Lymph node metastases, including some distant and uncommon lymph node metastases, are listed in table 2.

Thirty-three patients (25%) had 49 bone metastases. All the bone metastases showed osteolytic destruction with positive ^18^F-FDG uptake. Five patients had widespread osseous metastases. The most frequent bone metastases were thoracic vertebrae, ilium, and ribs in 9 (6.8%), 9 (6.8%) and 5 (3.8%) patients, respectively.

Lung metastases were found in 28 (21.2%) patients. The pulmonary metastatic lesions were characterized by a dense nodule on CT with positive ^18^F-FDG uptake.

Peritoneal metastases/abdominal wall metastases were identified in 7 (5.3%) patients. Five (3.8%) patients had metastases either in the pleura or in the soft tissues of thoracic wall.

Extrahepatic metastases in the gastrointestinal tract were depicted in 10 (7.6%) patients. Specifically, metastatic lesions were found in the head of pancreas in 5 (3.8%), stomach in 4 (3.0%) and duodenum in 1 (0.8%) patients.

There were 2 (1.5%) patients with metastatic sites in the limbs. These lesions, one in left forearm and one in right proximal thigh, were characterized as soft tissue masses with positive ^18^F-FDG uptake which had not invaded into bones.

The remaining uncommon metastases which were seldomly reported were spleen (3 patients), adrenal glands and diaphragm (2 patients each), brain, bladder and uterus (1 patient each).

### Prognosis of HCC patients with extrahepatic metastasis

The risk factors of overall survival for all the patients were analyzed using 13 clinical variables. Table 3 shows the results of the univariate and multivariate analyses. Four risk factors(prothrombin time, Child-Pugh grade, portal/hepatic vein invasion and lymph node metastasis) were associated with overall survival on univariate analysis (*P* = 0.039, 0.012, 0.042 and 0.036, respectively). Total bilirubin at a *p* value of 0.051 had almost reached to become a significant risk factor of overall survival. On multivariate analysis lymph node metastasis remained as the only independent risk factor of overall survival (*P* = 0.040).

**Table 3:** Univariate and multivariate analyses of predictors of survival for the HCC patients with extrahepatic metastasis.

Variables	Hazard Ratio	95% CI	*P* value
Univariate analysis			
Sex (Male *vs.* Female)	1.137	0.630∼2.053	0.669
Age (≤48 *vs.* >48 years)	1.098	0.681∼1.772	0.701
PS(0∼1 *vs.* 2∼4)	1.876	0.793∼2.638	0.132
Serum albumin (≤35 *vs.* >35 g/L)	0.745	0.539∼1.563	0.401
Total bilirubin(≤21 *vs.* >21 µmol/L)	1.683	0.997∼2.843	0.051
Prothrombin time (≤12.8 *vs.* >12.8 s)	1.659	1.026∼2.682	0.039
AFP level (≤400 *vs.* >400 ng/mL)	1.047	0.620∼1.767	0.864
Child–Pugh grade(A *vs.* B,C)	2.019	1.165∼3.498	0.012
Portal/hepatic vein invasion(Yes *vs.* No)	1.724	1.016∼2.437	0.042
CLIP Score(0∼3 *vs.* 4∼6)	1.129	0.768∼2.139	0.568
Site (lymph node *vs.* others)*	1.671	1.018∼2.703	0.036
Site (bone *vs.* others)*	0.894	0.507∼1.575	0.697
Site (lung *vs.* others)*	1.001	0.563∼1.779	0.998
Multivariate analysis			
Site (lymph node *vs.* others)*	1.878	1.025∼3.161	0.040

*Site: site of extrahepatic metastases.

A comparison of the clinicopathological data between patients with or without lymph node metastasis revealed that the group of patients with lymph node metastasis had a significantly higher total bilirubin (*P* = 0.009), and a significantly lower alpha fetoprotein (AFP) level (*P* = 0.035). As the tumor-node-metastasis (TNM) staging is also based on lymph node metastasis, it is not surprising that there were significantly more patients with TNM stage IV in the group of patients with lymph node metastasis. There were no significant differences between the two groups in sex, age, etiology, performance status, serum albumin, prothrombin time, Child–Pugh grade, portal/hepatic vein invasion, Cancer of the liver Italian program (CLIP) Score and Barcelona clinic liver cancer (BCLC) staging (Table 4).

**Table 4:** Clinical profile of HCC patients with or without lymphatic metastasis.

Variables	Patients with lymphatic metastasis (n = 72)	Patients without lymphatic metastasis (n = 60)	*P* value
Sex(Male/Female)	56/16	48/12	0.756
Age (years) *	48(26∼86)	48(26∼73)	0.833
Etiology(HBV/others)	56/16	49/11	0.667
PS(0/1/2/3/4)	42/25/4/1/0	27/27/4/1/1	0.127
Serum albumin (g/L) *	39.2(24.1∼50.3)	39.9(24.0∼54.6)	0.802
Total bilirubin(µmol/L) *	16.9(7.1∼338.2)	14.9(5.6∼255.4)	0.009
Prothrombin time (s) *	12.8(11.2∼22.5)	12.6(10.4∼23.9)	0.438
AFP level (ng/mL) *	9.0(0.2∼1123156.0)	79.5(1.89∼60000.0)	0.035
Child–Pugh grade(A/B/C)	56/14/2	52/6/2	0.490
Portal/hepatic vein invasion	32	26	0.898
CLIP Score(0∼3 *vs.* 4∼6)	46/31	41/14	0.074
TNM stage(III/IV)	0/72	5/55	0.018
BCLC stage(C/D)	69/3	57/3	1.000

*Value expressed in median with range in parentheses.

As far as possible, active treatment to control intrahepatic disease for patients with extrahepatic metastases was carried out. These treatments included liver resection (n = 28), radio-frequency ablation (RFA) (n = 15), trans-hepatic arterial chemoembolization (TACE) (n = 10) and supportive care (n = 79). There were no significant differences in the incidences of extrahepatic metastatic sites between the patients who were treated with resection/ablation of intrahepatic lesions (liver resection n = 28, RFA n = 15) when compared with those who were treated with regional therapy (TACE n = 10) or supportive care (n = 79). Sixteen patients who had lymph node metastases received liver resection (other patients had metastases at other sites). In 12 patients lymphadenectomy was also carried out. Histopathological study on the lymph nodes showed positivity in 11 patients.

**Figure 1:**
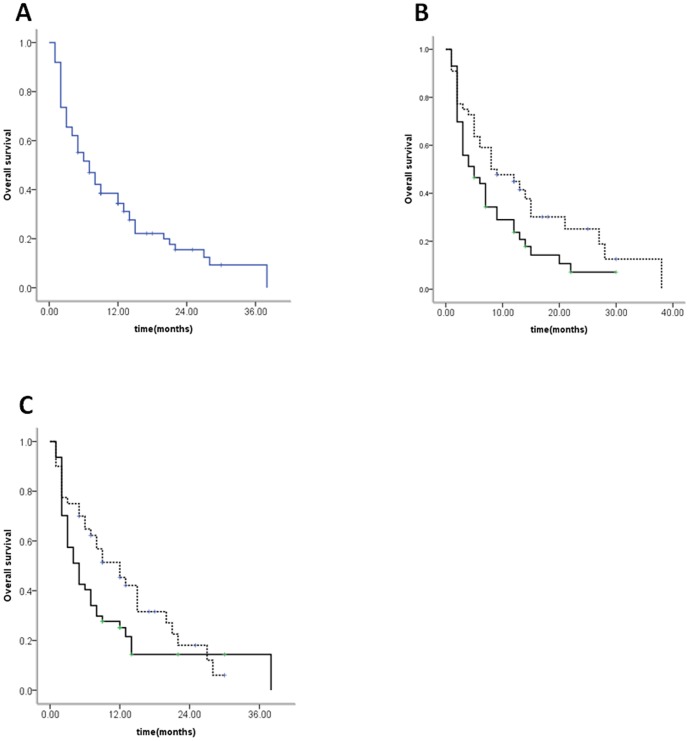
Survival curves of HCC patients. (A) Overall survival of all the HCC patients after initial diagnosis of extrahepatic metastasis. The cumulative survival rates at 12 and 36 months were 34.4% and 9.3%, respectively. (B) Comparison of survival rates between patients with or without active intrahepatic lesions (solid line: with active intrahepatic lesions (n = 89), dash line: without active intrahepatic lesions (n = 43), P = 0.037). (C) Comparison of survival rates between patients with or without lymph node metastases (solid line: with lymph node metastases, dash line: without lymph node metastases, *P* = 0.036).

The cumulative survival rates at 1- and 3-years after diagnosis of extrahepatic metastases of HCC were 34.4% and 9.3%, respectively (Figure 1A). The median survival was 7 months (range 1∼38 months). As the intra-hepatic lesions had been actively controlled using partial hepatectomy n = 28, or RFA n = 15, 43 patients had no active intra-hepatic lesion but they had only active extrahepatic lesions. The median survival time for patients with active intra-hepatic and extrahepatic lesions (treated by TACE or supportive care, n = 89) was 5 months (range 1∼30 months), while those with only active extrahepatic metastases (treated by partial hepatectomy or RFA) was 8 months (range 1∼38 months). The prognosis of HCC patients with active intra-hepatic lesions and extrahepatic metastases was significantly poorer than those with only active extrahepatic metastases (*P* = 0.037) (Figure 1B). The median survival time for patients with or without lymphatic metastasis were 5 months (range 1∼38 months) and 12 months (range 1∼30 months), respectively (Figure 1C). There was a significant difference between the two groups for overall survival (*P* = 0.036).

Four patients received sorafenib and they all died within 6 months of treatment. There was no patient with an isolated secondary which could be resected with an intention to cure. There were only five patients who received antiviral therapy with nucleoside analogues for a high load of hepatitis B virus (HBV-DNA>10^4^).

## Discussion

HCC is one of the most aggressive neoplasm, and extrahepatic metastases is common at the time of initial diagnosis [18], [19]. In the present study, the most frequent extrahepatic metastatic sites were lymph nodes, bone and lung. Survival analysis showed lymph node metastasis to be the only risk factor of overall survival indicating that HCC patients with lymph node metastasis had a very poor prognosis.

Previous reports have also showed that lung, abdominal lymph nodes, and bone to be the most common sites of extrahepatic metastases of HCC [15]–[17]. Several clinical studies and an autopsy study reported the incidences of HCC metastases to lung (n = 18, or 55%), to lymph nodes (n = 27, or 53%), to bone (n = 6, or 38%), and to adrenal glands (n = 8, or 15.4%) of patients with extrahepatic metastases. Sun et al indicated that the incidence of lymph node metastasis was 5.1% (49/968) in a study which evaluated the value of routine lymphadenectomy [20]. However, in this study, the tumors were resectable and the lymph nodes studied were loco-regional lymph nodes. According to the study performed by the Liver Cancer Study Group of Japan, 417 of 1374 patients had lymph node metastasis (30.3%) in autopsy series [21]. All these reported series were based on conventional workup using CT, MRI, chest X-ray and bone scintigraphy, or by histopathological examination of surgically resected specimens or by autopsy. Several recently published reports which compared PET/CT with conventional medical imaging in the detection of extrahepatic metastasis of HCC concluded that ^18^F-FDG PET/CT was a better and non-invasive diagnostic tool for the detection of extrahepatic metastases. In studies on ^18^F-FDG PET/CT, only non-diabetic patients with normal range glucose should be chosen. The ^18^fluorine-fluorodeoxyglucose (^18^F-FDG) is a glucose analog which has been widely used in tumor imaging. The tumoral uptake of ^18^F-FDG is based upon enhanced glycolysis. Following administration, ^18^F-FDG is phosphorylated and trapped intracellularly which forms the basis of PET imaging. An important mechanism to transport ^18^F-FDG into the tumor cell is based upon the action of glucose transporter proteins. Furthermore, highly active hexokinase bound to tumor mitochondria helps to trap ^18^F-FDG into the cells. ^18^F-FDG uptake may be due to the relative hypoxia in tumor masses, which activate the anaerobic glycolytic pathway. In spite of these processes, ^18^F-FDG uptake is relatively non-specific since all living cells need glucose. The clinical application of ^18^F-FDG imaging is, therefore, recommended in carefully selected patients. Kim et al. showed ^18^F-FDG PET/CT to be more sensitive in the detection of extrahepatic lesions, and recommended it to be used as a screening tool for follow-up after liver transplantation (LT) for HCC [8]. ^18^F-FDG PET/CT has the ability to provide additional information beyond that can be provided by conventional imaging techniques, and contributes to the clinical management of HCC patients. Our study showed that lymph nodes were the most frequent sites of metastasis and the incidence of lymph node metastasis was beyond 50%, supporting that ^18^F-FDG PET/CT has a higher sensitivity than conventional imaging techniques to detect metastasis as some lymph node metastases were negative on conventional imaging but were positive on ^18^F-FDG PET/CT. The most frequent site of lymph node metastasis was retroperitoneal lymph nodes, but not porta hepatis lymph nodes which are the regional lymph nodes draining the liver. On survival analyses, HCC patients with lymph node metastasis had a significantly worse overall survival than patients without lymph node metastasis. This result agrees with the results of the studies which have previously been reported. Lee et al. reported that lymph node metastasis was a poor prognostic factor for HCC based on the analysis of 25 operable patients diagnosed with lymph node metastasis after matching patients with and without lymph node metastasis [22]. HCC with lymph node metastasis tended to be HCC of the infiltrating type, with large tumor size (>5 cm), presence of microvascular invasion, and bad histological grade. These risk factors shortened the overall survival of the patients [23]–[25]. In our study, the total bilirubin was much higher in the group with lymph node metastasis than in the group without. A higher total bilirubin means poorer liver function. According to the Barcelona Clinic Liver Cancer staging classification, HCC with lymph node metastasis represents an advanced tumor stage. The same is true for the TNM staging system of HCC.

In the present study, the prognosis of HCC patients with active intra-hepatic lesions and extrahepatic metastases was significantly poorer than those with only active extrahepatic metastases. For the rare patient with an isolated lung metastases, if the intra-hepatic lesion can be controlled by surgery, pulmonary metastasectomy can provide an opportunity for disease control and long-term survival [26], [27]. Moreover, some studies reported that the prognosis of patients with intra-hepatic recurrence before lung metastases was worse than that for patients with first metastases in the lung [28], [29]. The majority of HCC patients with extrahepatic metastasis do not die of metastatic dissemination but rather die of hepatic failure due to progression of intra-hepatic HCC [15], [16]. Therefore, the majority of patients with HCC with extrahepatic metastasis should undergo locoregional treatment for the intra-hepatic HCC. In other words, the treatment of intra-hepatic HCC is almost always required to improve survival when hepatic function and extent of disease permit. It is still uncertain whether patients with lymph node metastasis could benefit from lymphadenectomy. Previous studies have shown lymphadenectomy produced little benefits but it increased the risk of postoperative complications [20], [22]. In our study, there were 33 patients who had two or more metastatic sites and there was no evidence to show the different metastatic sites to be mutually exclusive of each other.

There are some limitations in the present study. First, the population size of this study is relatively small, although this is the largest study from a single center. A multicenter study is needed to include more patients into such a type of study. Second, not all extrahepatic metastases had histopathologic confirmation, although the diagnosis was made by comprehensive assessment based on clinical characteristics, imaging and ^18^F-FDG PET/CT. As ^18^F-FDG PET/CT is currently the most sophisticated technique to detect metastases, despite the possibility of false positivity, it is still commonly used as a standard to diagnose extrahepatic metastasis. A systematic review and meta-analysis published recently showed the pooled estimates of sensitivity, specificity, positive likelihood ratio(LR+), and negative likelihood ratio(LR−) of ^18^F-FDG PET/CT in the detection of metastatic HCC were 76.6%, 98.0%, 14.68, and 0.28, respectively [14].

In conclusion, our present study indicated that lymph node metastasis was the most frequent site of extrahepatic metastases of primary HCC. Lymph node metastasis was the main risk factor of overall survival in HCC patients with extrahepatic metastasis. Effective treatment for intra-hepatic lesions would benefit HCC patients with extrahepatic metastases.

## References

[1] Jemal A, Bray F, Center MM, Ferlay J, Ward E, et al (2011) Global cancer statistics. CA Cancer J Clin 61: 69–90. 21296855 10.3322/caac.20107

[2] Katyal S, Oliver JH 3rd, Peterson MS, Ferris JV, Carr BS, et al (2000) Extrahepatic metastases of hepatocellular carcinoma. Radiology 216: 698–703. 10966697 10.1148/radiology.216.3.r00se24698

[3] Shuto T, Hirohashi K, Kubo S, Tanaka H, Yamamoto T, et al (2001) Treatment of adrenal metastases after hepatic resection of a hepatocellular carcinoma. Dig Surg 18: 294–297. 11528139 10.1159/000050155

[4] Si MS, Amersi F, Golish SR, Ortiz JA, Zaky J, et al (2003) Prevalence of metastases in hepatocellular carcinoma: risk factors and impact on survival. Am Surg 69: 879–885. 14570367

[5] El-Serag HB, Rudolph KL (2007) Hepatocellular carcinoma: epidemiology and molecular carcinogenesis. Gastroenterology 132: 2557–2576. 17570226 10.1053/j.gastro.2007.04.061

[6] Yoon KT, Kim JK, Kim do Y, Ahn SH, Lee JD, et al (2007) Role of 18F-fluorodeoxyglucose positron emission tomography in detecting extrahepatic metastasis in pretreatment staging of hepatocellular carcinoma. Oncology 72 Suppl 1104–110. 18087190 10.1159/000111715

[7] Llovet JM, Real MI, Montana X, Planas R, Coll S, et al (2002) Arterial embolisation or chemoembolisation versus symptomatic treatment in patients with unresectable hepatocellular carcinoma: a randomised controlled trial. Lancet 359: 1734–1739. 12049862 10.1016/S0140-6736(02)08649-X

[8] Kim YK, Lee KW, Cho SY, Han SS, Kim SH, et al (2010) Usefulness 18F-FDG positron emission tomography/computed tomography for detecting recurrence of hepatocellular carcinoma in posttransplant patients. Liver Transpl 16: 767–772. 20517911 10.1002/lt.22069

[9] Kawaoka T, Aikata H, Takaki S, Uka K, Azakami T, et al (2009) FDG positron emission tomography/computed tomography for the detection of extrahepatic metastases from hepatocellular carcinoma. Hepatol Res 39: 134–142. 19208034 10.1111/j.1872-034X.2008.00416.x

[10] He YX, Guo QY (2008) Clinical applications and advances of positron emission tomography with fluorine-18-fluorodeoxyglucose (18F-FDG) in the diagnosis of liver neoplasms. Postgrad Med J 84: 246–251. 18508981 10.1136/pgmj.2007.066589

[11] Wudel LJ Jr, Delbeke D, Morris D, Rice M, Washington MK, et al (2003) The role of [18F]fluorodeoxyglucose positron emission tomography imaging in the evaluation of hepatocellular carcinoma. Am Surg 69: 117–124 discussion 124–116. 12641351

[12] Ho CL, Chen S, Yeung DW, Cheng TK (2007) Dual-tracer PET/CT imaging in evaluation of metastatic hepatocellular carcinoma. J Nucl Med 48: 902–909. 17504862 10.2967/jnumed.106.036673

[13] Sugiyama M, Sakahara H, Torizuka T, Kanno T, Nakamura F, et al (2004) 18F-FDG PET in the detection of extrahepatic metastases from hepatocellular carcinoma. J Gastroenterol 39: 961–968. 15549449 10.1007/s00535-004-1427-5

[14] Lin CY, Chen JH, Liang JA, Lin CC, Jeng LB, et al (2012) 18F-FDG PET or PET/CT for detecting extrahepatic metastases or recurrent hepatocellular carcinoma: a systematic review and meta-analysis. Eur J Radiol 81: 2417–2422. 21899970 10.1016/j.ejrad.2011.08.004

[15] Natsuizaka M, Omura T, Akaike T, Kuwata Y, Yamazaki K, et al (2005) Clinical features of hepatocellular carcinoma with extrahepatic metastases. J Gastroenterol Hepatol 20: 1781–1787. 16246200 10.1111/j.1440-1746.2005.03919.x

[16] Ochiai T, Ikoma H, Okamoto K, Kokuba Y, Sonoyama T, et al (2012) Clinicopathologic features and risk factors for extrahepatic recurrences of hepatocellular carcinoma after curative resection. World J Surg 36: 136–143. 22051887 10.1007/s00268-011-1317-y

[17] Tanaka K, Shimada H, Matsuo K, Takeda K, Nagano Y, et al (2008) Clinical features of hepatocellular carcinoma developing extrahepatic recurrences after curative resection. World J Surg 32: 1738–1747. 18463920 10.1007/s00268-008-9613-x

[18] Chan KM, Yu MC, Wu TJ, Lee CF, Chen TC, et al (2009) Efficacy of surgical resection in management of isolated extrahepatic metastases of hepatocellular carcinoma. World J Gastroenterol 15: 5481–5488. 19916180 10.3748/wjg.15.5481 PMC2778106

[19] Taketomi A, Toshima T, Kitagawa D, Motomura T, Takeishi K, et al (2010) Predictors of extrahepatic recurrence after curative hepatectomy for hepatocellular carcinoma. Ann Surg Oncol 17: 2740–2746. 20411432 10.1245/s10434-010-1076-2

[20] Sun HC, Zhuang PY, Qin LX, Ye QH, Wang L, et al (2007) Incidence and prognostic values of lymph node metastasis in operable hepatocellular carcinoma and evaluation of routine complete lymphadenectomy. J Surg Oncol 96: 37–45. 17345597 10.1002/jso.20772

[21] Primary liver cancer in Japan. Clinicopathologic features and results of surgical treatment. Ann Surg 211: 277–287. 2155591 PMC1358432

[22] Lee CW, Chan KM, Lee CF, Yu MC, Lee WC, et al (2011) Hepatic resection for hepatocellular carcinoma with lymph node metastasis: clinicopathological analysis and survival outcome. Asian J Surg 34: 53–62. 21723467 10.1016/S1015-9584(11)60020-1

[23] Xiaohong S, Huikai L, Feng W, Ti Z, Yunlong C, et al (2010) Clinical significance of lymph node metastasis in patients undergoing partial hepatectomy for hepatocellular carcinoma. World J Surg 34: 1028–1033. 20174806 10.1007/s00268-010-0400-0

[24] Ercolani G, Grazi GL, Ravaioli M, Grigioni WF, Cescon M, et al (2004) The role of lymphadenectomy for liver tumors: further considerations on the appropriateness of treatment strategy. Ann Surg 239: 202–209. 14745328 10.1097/01.sla.0000109154.00020.e0 PMC1356213

[25] Uka K, Aikata H, Takaki S, Shirakawa H, Jeong SC, et al (2007) Clinical features and prognosis of patients with extrahepatic metastases from hepatocellular carcinoma. World J Gastroenterol 13: 414–420. 17230611 10.3748/wjg.v13.i3.414 PMC4065897

[26] Lee CY, Bae MK, Park IK, Kim DJ, Lee JG, et al (2010) Surgical resection for pulmonary metastasis from hepatocellular carcinoma: analysis of prognosis in relation to primary control. J Surg Oncol 101: 239–243. 20127898 10.1002/jso.21487

[27] Sakamoto M, Murakawa T, Kitano K, Murayama T, Tsuchiya T, et al (2010) Resection of solitary pulmonary lesion is beneficial to patients with a history of malignancy. Ann Thorac Surg 90: 1766–1771. 21095305 10.1016/j.athoracsur.2010.07.054

[28] Yoon YS, Kim HK, Kim J, Choi YS, Shim YM, et al (2010) Long-term survival and prognostic factors after pulmonary metastasectomy in hepatocellular carcinoma. Ann Surg Oncol 17: 2795–2801. 20517683 10.1245/s10434-010-1073-5

[29] Kitano K, Murayama T, Sakamoto M, Nagayama K, Ueno K, et al (2012) Outcome and survival analysis of pulmonary metastasectomy for hepatocellular carcinoma. Eur J Cardiothorac Surg 41: 376–382. 21727012 10.1016/j.ejcts.2011.05.052

